# It’s a small world: *Sinningia* double flower cultivars share the same *GLOBOSA1* allele

**DOI:** 10.1093/plcell/koaf031

**Published:** 2025-03-06

**Authors:** Andrew C Willoughby

**Affiliations:** Assistant Features Editor, The Plant Cell, American Society of Plant Biologists; Department of Biology, Duke University, Durham, NC 27713, USA

*Sinningia* is a genus in the largely tropical Gesneriaceae family that includes popular ornamental plants. In new work, **Xia Yang and colleagues (Yang et al. 2025)** characterize the genetic nature of a desirable horticultural trait, double flowers, and track the shared origin of this trait—considered a result of hybridization—in a collection of 15 *Sinningia* cultivars. There are limited resources available for studying plants in the Gesneriaceae. Therefore, the authors took advantage of transformation tools they previously developed for a different Gesneriaceae species (Liu et al. 2014). They found that an insertion in the promoter of a petal identity gene contains regulatory elements that cause ectopic expression and the double flower phenotype in *Sinningia* cultivars.

Double flowers result from the conversion of a floral organ into petals; in double “hose-in-hose” (*hih*) *Sinningia* flowers, the sepals are converted into an additional whorl of petals (Figure). The authors analyzed the expression of floral organ identity genes in 2 *hih* cultivars as well as the wild-type *S. eumorpha* and determined that petal identity genes such as *GLOBOSA1* (*GLO1*) and *GLO2* are more broadly expressed in *hih* plants. Crosses between *hih* cultivars and *S. eumorpha* demonstrated that the double flower phenotype is dominant. Using these F1 plants, the authors found that only the *GLO1* genotype is linked to the double flower phenotype. They investigated a 225-bp insertion upstream of the *GLO1* gene in *hih* plants that resembles a *hAT-*like miniature inverted-repeat transposable element (*hAT*-MITE) and identified several transcription factor binding motifs in this insertion. Transient expression assays in *Nicotiana benthamiana* demonstrated that the *hih* insertion is capable of driving *GLO1* expression, potentially explaining the ectopic expression of *GLO1* in *hih* plants. Transgenic *S. eumorpha* has not been reported, and there are very few examples of transgenic Gesneriaceae (Liu et al. 2014; Ghorbanzade and Ahmadabadi 2015; Nishii et al. 2020; Liu et al. 2021). To demonstrate the sufficiency of the *hAT*-MITE*-*containing *GLO1* allele to drive the *hih* phenotype, Yang et al. expressed both *hih* alleles in transgenic *Chirita pumila*, a species the authors previously proposed as a model for Gesneriaceae research (Liu et al. 2014, 2021), and showed that the *hih* phenotype is recapitulated only when the insertion is present in the *GLO1* promoter (Figure).

**Figure.**
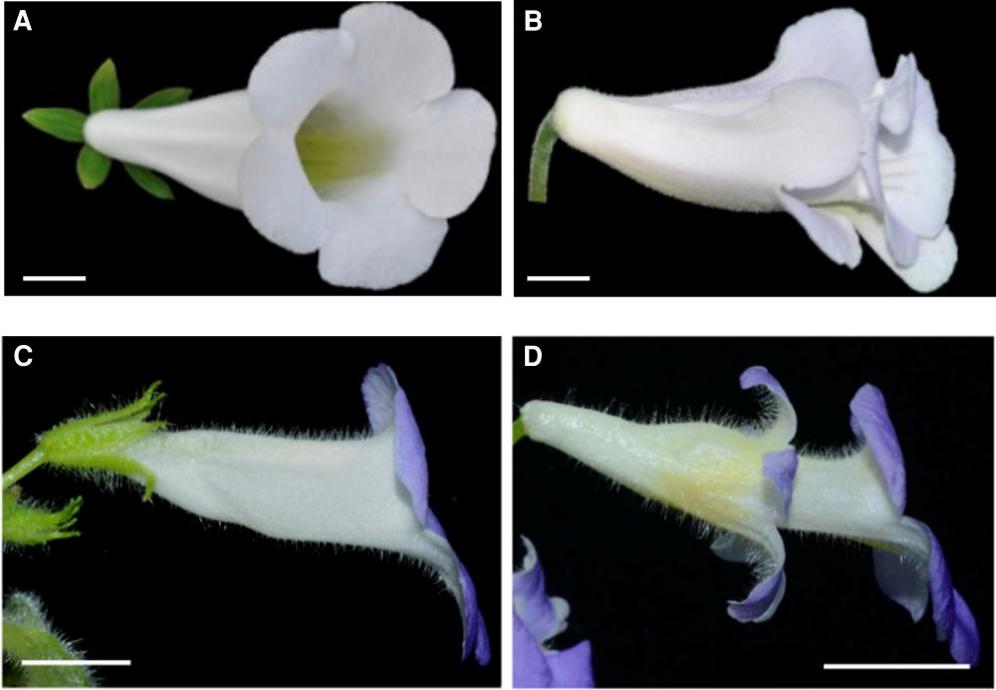
A *GLOBOSA* allele underlies *hih* flowers in *Sinningia*. **A)** Wild-type *S. eumorpha*. **B)**  *Sinnginia hih2* cultivar with *hih* phenotype. **C, D)** Stable transformation of the dominant *GLO1 hih* allele into wild-type *C. pumila* generates the *hih* phenotype, confirming the causative nature of this allele in *Sinningia hih* cultivars. Flowers of wild-type *C. pumila* (C) and transformant carrying a dominant *hih GLO1* allele (D). A-B from Dr. Xia Yang with permission. C-D adapted from Yang et al. (2025), Figure 6. Scale bars, 1 cm.

The authors tracked the origin of the *hih* phenotype in 15 *Sinningia* cultivars and found that they contain a *hAT-MITE* sequence identical to one in *S. cardinalis*, although in *S. cardinalis* it is not inserted into *GLO1*. Because activation of *hAT*-MITEs can be caused by hybridization and stresses associated with domestication, the authors hypothesize that a hybridization event with *S. cardinalis* resulted in the insertion of the *S. cardinalis hAT-MITE* into *GLO1* to create the *hih* allele in F1 plants. Descendants of these plants were crossed into a variety of other cultivars to generate new *hih* plants by introgression of the same allele.

## References

[koaf031-B1] Ghorbanzade Z, Ahmadabadi M. Stable transformation of the *Saintpaulia ionantha* by particle bombardment. Iran J Biotechnol. 2015:13(1):11–16. 10.15171/ijb.1037 28959276 PMC5434982

[koaf031-B2] Liu B-L, Yang X, Liu J, Dong Y, Wang Y-Z. Characterization, efficient transformation and regeneration of *Chirita pumila* (Gesneriaceae), a potential evo-devo model plant. Plant Cell Tiss Organ Cult. 2014:118(2):357–371. 10.1007/s11240-014-0488-2

[koaf031-B3] Liu J, Wang J-J, Wu J, Wang Y, Liu Q, Liu F-P, Yang X, Wang Y-Z. An optimized transformation system and functional test of *CYC*-like TCP gene *CpCYC* in *Chirita pumila* (Gesneriaceae). Int J Mol Sci. 2021:22(9):4544. 10.3390/ijms22094544 33925272 PMC8123712

[koaf031-B4] Nishii K, Fei Y, Hudson A, Möller M, Molnar A. Virus-induced gene silencing in Streptocarpus rexii (Gesneriaceae). Mol Biotechnol. 2020:62(6–7):317–325. 10.1007/s12033-020-00248-w 32146689

[koaf031-B5] Yang X, Liu Q, Wang M-M, Wang X-Y, Han M-Q, Liu F-P, Lü T-F, Liu J, Wang Y-Z. A single dominant *GLOBOSA* allele accounts for repeated origins of hose-in-hose flowers in *Sinningia* (Gesneriaceae). Plant Cell. 2025:37(1):koae283. 10.1093/plcell/koae283 PMC11663566 39422240

